# The Efficacy of Generating Three Independent Anti-HIV-1 siRNAs from a Single U6 RNA Pol III-Expressed Long Hairpin RNA

**DOI:** 10.1371/journal.pone.0002602

**Published:** 2008-07-02

**Authors:** Sheena Saayman, Samantha Barichievy, Alexio Capovilla, Kevin V. Morris, Patrick Arbuthnot, Marc S. Weinberg

**Affiliations:** 1 Antiviral Gene Therapy Research Unit, Department of Molecular Medicine and Haematology, University of Witwatersrand, Johannesburg, South Africa; 2 HIV Pathogenesis Lab, Department of Molecular Medicine and Haematology, University of Witwatersrand, Johannesburg, South Africa; 3 Department of Molecular and Experimental Medicine, The Scripps Research Institute, La Jolla, California, United States of America; National Institute for Communicable Diseases, South Africa

## Abstract

RNA Interference (RNAi) effectors have been used to inhibit rogue RNAs in mammalian cells. However, rapidly evolving sequences such as the human immunodeficiency virus type 1 (HIV-1) require multiple targeting approaches to prevent the emergence of escape variants. Expressed long hairpin RNAs (lhRNAs) have recently been used as a strategy to produce multiple short interfering RNAs (siRNAs) targeted to highly variant sequences. We aimed to characterize the ability of expressed lhRNAs to generate independent siRNAs that silence three non-contiguous HIV-1 sites by designing lhRNAs comprising different combinations of siRNA-encoding sequences. All lhRNAs were capable of silencing individual target sequences. However, silencing efficiency together with concentrations of individual lhRNA-derived siRNAs diminished from the stem base (first position) towards the loop side of the hairpin. Silencing efficacy against HIV-1 was primarily mediated by siRNA sequences located at the base of the stem. Improvements could be made to first and second position siRNAs by adjusting spacing arrangements at their junction, but silencing of third position siRNAs remained largely ineffective. Although lhRNAs offer advantages for combinatorial RNAi, we show that good silencing efficacy across the span of the lhRNA duplex is difficult to achieve with sequences that encode more than two adjacent independent siRNAs.

## Introduction

RNA Interference (RNAi) is a highly conserved biological pathway in eukaryotes where gene silencing is mediated by a double-stranded RNA (dsRNA) trigger [1]. Exploitation of the RNAi pathway has lead to fundamental new tools for genetics research and for sequence-specific therapeutic approaches aimed at suppressing rogue cellular genes or viral-associated RNAs. RNAi has traditionally been induced in mammalian cells through the exogenous introduction of synthetic short interfering RNAs (siRNAs) [2], or through the use of RNA Pol III or Pol II gene constructs which express 21–29 bp short hairpin RNAs (shRNAs) [3]–[6]. Expressed short hairpins resemble pre-microRNAs (pre-miRNAs), which are part of the endogenous microRNA (miRNA) pathway [7], [8]. The targeting of highly mutable sequences, such as genomic and sub-genomic RNAs from infectious agents, remains a significant hurdle for the use of RNAi-based therapeutics. In particular, the human immunodeficiency virus type 1 (HIV-1), which replicates using an error-prone reverse transcriptase, has been shown to escape the silencing effects of shRNAs. Resistant viral variants emerge easily in cell culture experiments, even when targeting highly conserved sequences [9]–[11]. Effective targeting of rapidly evolving targets requires a combinatorial approach, which is analogous to Highly Active Antiretroviral Therapy (HAART) [reviewed by [12], [13]].

The targeting of many sites simultaneously using RNAi has been attempted with multiple shRNA expression units, where each unit is expressed from a RNA Pol III promoter [14]–[16] or RNA Pol II promoter [17]. Similarly, concatenated miRNA mimics expressed from a single RNA Pol II promoter have been shown to suppress simultaneously up to three separate target sequences [6], [18], [19]. Although RNAi-mediated silencing in lower eukaryotes can be achieved efficiently by introducing precursor dsRNAs comprising more than 150 base pairs (bp), intracellular presence of dsRNA of greater than 30 bp leads to a strong innate immunostimulatory response, which is mediated by dsRNA-activating protein kinase (PKR) and 2′–5′ oligoadenylate synthetase [20]. A re-evaluation of long dsRNA greater than 30 bp in mammalian cells has shown that safe and effective gene-specific silencing can be achieved when dsRNA is expressed from DNA-based expression cassettes [21]–[26]. Although a complete characterization of how intracellular dsRNAs are discriminated remains to be established, intracellularly expressed dsRNA seem capable of evading cytoplasmic activators of the type 1 interferon response [27], [28]. A natural potential advantage of longer dsRNAs is that processing by the RNAse III endonuclease Dicer theoretically allows for the generation of multiple siRNAs, providing a mechanism of combinatorial targeting of rapidly-evolving RNAs. The silencing caused by lhRNAs may also be more effective than that resulting from a single unique siRNA derived from an individual shorter (<30 bp) expressed shRNA.

Akashi et al. showed that (∼50 bp) long hairpin RNAs (lhRNAs) expressed from tRNA^Val^ and U6 RNA Pol III promoters generated multiple siRNAs [24]. We and other have shown that similar constructs were capable of suppressing Hepatitis B Virus (HBV) [26], Hepatitis C Virus (HCV) [29] and HIV-1 [30]–[33] targets. To date, lhRNAs capable of producing more than two independent siRNAs have only been used against contiguous target sequences. Since ∼60 bp hairpin RNAs should be capable of providing a substrate for at least three catalytic reactions involving Dicer, we have examined the possibility of introducing three distinct non-contiguous target sequences that, if processed by Dicer, are capable of generating highly-effective independent siRNA species. To determine the ability of targeting disparate regions in the HIV-1 genome, we generated a panel of ∼69 bp U6-lhRNA expression cassettes, each consisting of a different arrangement of three adjacent 21-mer putative siRNA sequences. The siRNA sequences chosen were previously characterized as highly effective anti-HIV-1 shRNAs targeted to Tat/Env, Tat/Rev and Vif open reading frames [4], [34]. We show that all combinations of lhRNAs were capable of significant knockdown against individual target sequences. However, silencing efficiency together with individual siRNA concentrations diminished from stem base to loop side along the length of the duplex. We present here a thorough characterisation of the efficacy of expressed lhRNAs designed to generate multiple siRNAs targeted to non-contiguous siRNA-susceptible regions of HIV-1.

## Results

### Design of anti HIV-1 lhRNA-expressing plasmids

**Figure 1:**
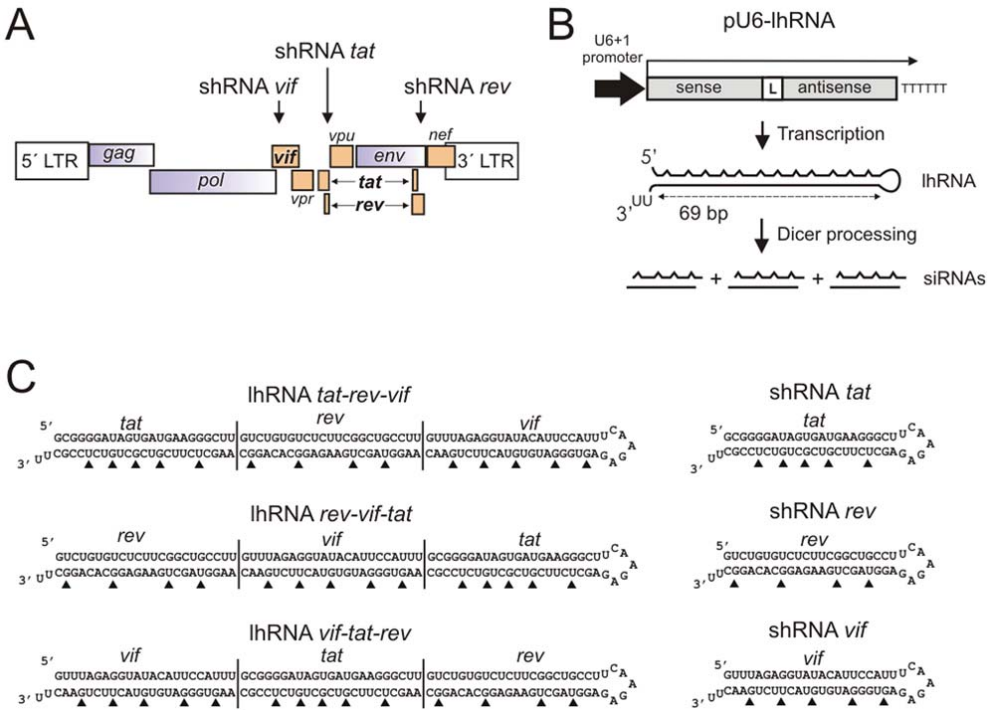
HIV-1 C subtype genome with sites targeted by lhRNA and shRNAs. A. Organization of HIV-1 subtype C genome indicating open reading frames (ORFs) together with the 5′ and 3′ long terminal repeats (LTRs). Arrows show the sites targeted by each of shRNA *vif*, shRNA *tat* and shRNA *rev*, as well as the lhRNAs. B. Schematic illustration of lhRNAs comprising 69 bp in the stem. G∶U pairings are indicated as corrugated sense strand. A sequence of 2 U residues that are derived from the transcription termination signal is shown. The intended mechanism of transcription and processing of the lhRNAs to form 3 anti HIV-1 siRNAs is illustrated. C. Sequences and predicted structure of lhRNAs and shRNAs. The order of the siRNA-encoding sequences within the lhRNAs is indicated along the extent of the duplex. G∶U and U∶G pairings are indicated with an arrowhead.

Three sites that have been previously shown to be effective for RNAi-mediated inhibition of HIV-1 were selected. These include shRNAs targeted to sites within two separate overlapping reading frames of the HIV-1 genome: Tat/Rev (*tat*) and Rev/Env (*rev*) [4]. The third site includes a sequence within the Vif open reading frame (*vif*) [34] (Fig. 1A). Long hairpin RNAs of approximately 69 bp (with a 5 nt terminal loop) were designed to be transcribed from a U6 RNA Pol III promoter such that three 21–23 bp siRNAs could potentially be generated by Dicer cleavage (Fig. 1B). LhRNA and shRNA expression cassettes were designed to encode siRNA precursors targeted to each of these 3 HIV-1 sites (Fig. 1C). G∶U wobble base pairs were included at regular intervals by adjusting sequences in the sense strand to facilitate propagation of the lhRNA-encoding cassettes in *E. coli*. To control for changes in strand bias, similar G∶U mismatches were made to each shRNA (Fig. 1C). These changes have previously been shown to have no impact on RNAi knockdown efficacy [24], [26] and may help in suppressing the innate immune response to dsRNA [24]. The lhRNAs were therefore intended to be capable of serving as substrates to form siRNAs against each of the *tat*, *rev* and *vif* HIV-1 targets. By targeting three viral sites simultaneously, the lhRNAs have a possible therapeutic benefit of limiting viral escape.

Although this is an important theoretical advantage, there is some evidence to suggest that Dicer processing of lhRNAs may not be equal across the span of the duplex [26], [33] and that Dicer favours the production of siRNAs generated from the hairpin stem base. Thus, to assess the importance of the position of the *tat*, *rev* and *vif* siRNA-encoding sequences within the anti HIV-1 lhRNAs, their efficacy when ordered as first, second or third within the stem duplex was assessed.

### Assessing anti HIV-1 efficacy of expressed lhRNA sequences in cell culture

**Figure 2:**
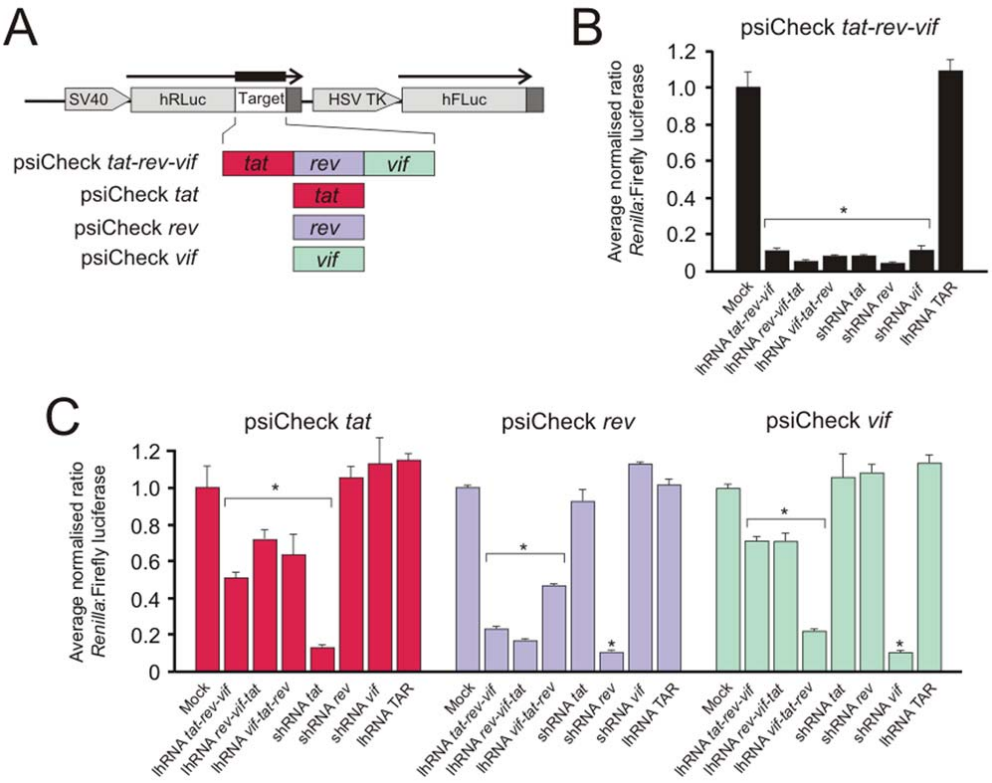
Knockdown of target-reporter fusion gene expression. A. psiCheck-derived vectors that include indicated HIV-1 target sequences inserted downstream of the *Renilla* luciferase ORF. The control Firefly luciferase cassette, present on the same plasmid, is also shown. Both cassettes are under control of constitutively active transcription regulatory elements: Herpes simplex virus thymidine kinase (HSV TK) and Simian virus 40 (SV40) promoters. B. Average normalized ratios of the *Renilla*∶Firefly luciferase activity when cells were transfected with psiCheck *tat*-*rev*-*vif* dual luciferase reporter plasmid together with lhRNA- or shRNA-encoding plasmid vectors. C. Average normalized ratios of the *Renilla*∶Firefly luciferase activity when cells were transfected with psiCheck *tat*, psiCheck *rev* or psiCheck *vif* dual luciferase reporter plasmids together with lhRNA- or shRNA-encoding plasmid vectors. The average values from three independent transfection experiments, with standard deviations, are given (*, *p*<0.05, *t*-test, relative to mock transfected control).

Initially, to assess efficacy against HIV-1 *in vitro*, HEK293 cells were cotransfected with lhRNA-or shRNA-expressing vectors together with the dual luciferase psiCheck vector encoding a reporter/HIV-1 fusion gene (Fig. 2A). Four target vectors were generated, which each included *tat*, *rev*, *vif* or a combination of *tat-rev-vif* HIV-1 21-mer targets downstream of the *Renilla* luciferase open reading frame (ORF). Measurement of *Renilla*∶Firefly luciferase allowed convenient and accurate measurement of the *in situ* efficacy of the hairpin sequences. When using the luciferase reporter that includes all three HIV-1 targets (psiCheck *tat-rev-vif*), highly effective knockdown of approximately 90% was achieved for each of the lhRNA- and shRNA-encoding plasmids (Fig. 2B). When the *tat* sequence alone was inserted downstream of the *Renilla* luciferase reporter gene, the shRNA *tat* vector was capable of 90% inhibition of reporter gene expression, and as expected, the shRNA *rev* and shRNA *vif* vectors caused no decrease in reporter fusion gene activity (Fig. 2C). lhRNA expression cassettes diminished *Renilla-tat* gene activity by approximately 30–50% and lhRNA *tat-rev-vif* was the most efficient. When similar assessment was carried out on the psiCheck *rev* and psiCheck *vif* targets, the shRNAs induced specific silencing and lhRNAs with the siRNA-encoding sequences located at the base of the stem duplex were most efficient. A 63 bp lhRNA targeted to the HIV-1 TAR loop, showed no inhibitory activity against any of the targets. These data support our previous observations using lhRNAs to inhibit HBV [26] and HIV [31], [33] replication and indicate that there is a bias of silencing efficiency that diminishes from the base of the stem to loop side of the duplex RNA.

### Detection of processed antiviral hairpin sequences

**Figure 3:**
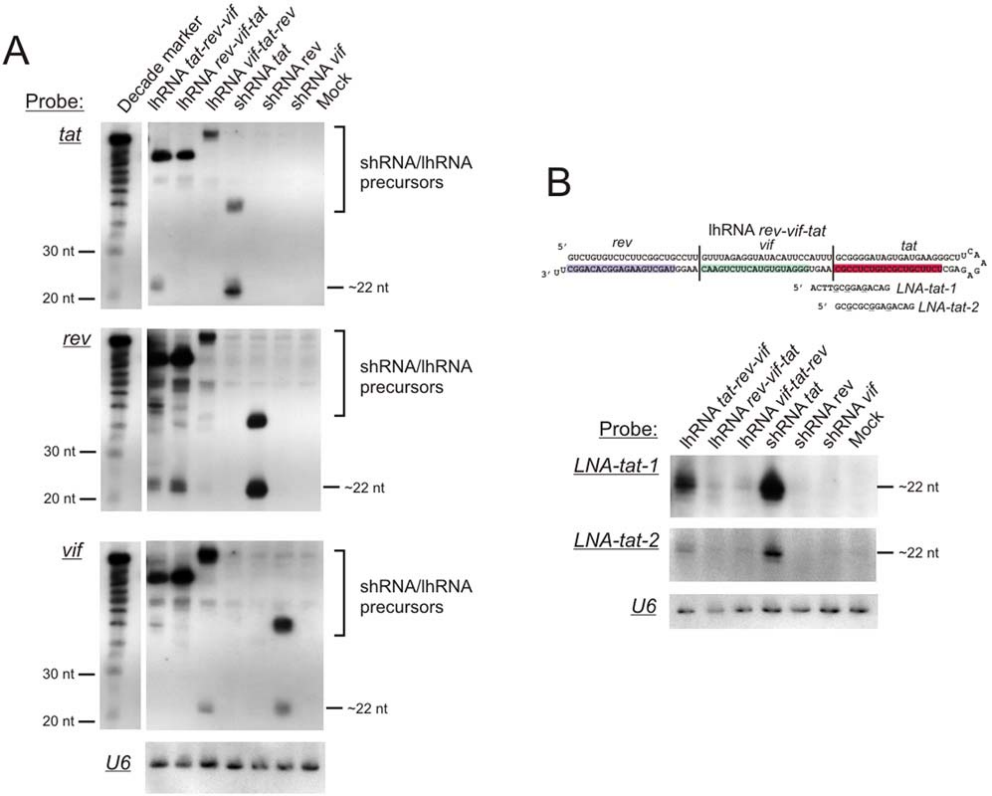
Northern blot analysis of RNA extracted from HEK293 cells that had been transfected with the indicated lhRNA and shRNA-expressing plasmids. A. A single blot was probed with an oligonucleotide that was complementary to putative *tat*, *rev* and *vif* guide sequences. B. The blot was probed with two 14-mer LNA oligonucleotides (LNA nucleotides underlined) which were complementary to the *rev* siRNA guide sequence and adjacent nucleotides as indicated in the illustration. Both blots (from A and B) were stripped and reprobed with an oligonucleotide complementary to U6 snRNA to control for equal RNA loading.

The spacing arrangement of each individual siRNAs within the long hairpin duplex is such that 4 “neutral” bases were placed between each 21-mer sequence. This arrangement was recently determined as optimal for two siRNAs placed within an extended shRNA [32]. To analyse primary transcripts and processed products of the anti HIV-1 hairpin expression cassettes a northern blot hybridisation was carried out. RNA was extracted from transfected HEK293 cells and Figure 3A shows the signals obtained after hybridisation to probes that were complementary to the putative mature processed *tat*, *rev* or *vif* siRNA guide strands. Mature products of each of the shRNA expression cassettes were detectable as bands of approximately 22–23 nt in size. Processing of the shRNA primary transcripts to produce siRNA appeared to occur more effectively than that of lhRNA expression cassettes for guide strands at the base of the duplex, which may be due to better recognition by Dicer for the shRNA than the lhRNA (Fig. 3A). The band representing precursors for construct lhRNA *vif-tat-rev* was larger than the precursor bands of the other two lhRNAs, suggesting that read-through transcription is occurring beyond the polyT termination signal. Detection with the vif probe suggests that the production of siRNAs were not impaired for lhRNA *vif-tat-rev* (at least in the first position). For the lhRNAs, guide strands derived from the duplex stem base region of the lhRNA expression cassettes were present in highest concentration, while those that originated from the second and third positions of the lhRNA stem were only detected for the lhRNA *rev-vif-tat* construct, in decreasing order of concentration. It is possible that the probes are not detecting the second or third siRNA because of misalignment due to differential Dicer processing. To investigate this further, we used two 14-mer locked-nucleic acid (LNA) probes, *LNA-tat-1* and *LNA-tat-2*, which were partially or fully complementary to the tat siRNA respectively (Fig. 3B). *LNA-tat-2* was designed to fully complement any siRNAs generated by three Dicer processing reactions that are 21-bases apart. The results show that only the *tat* siRNA guide derived from the shRNA *tat* or lhRNA *tat-rev-vif* could be detected by both probes, which supports the theory that there is a considerable drop in siRNA concentration as Dicer processes along the lhRNA duplex.

### Effect of spacing between siRNA-encoding sequences of the lhRNAs on silencing efficacy

**Figure 4:**
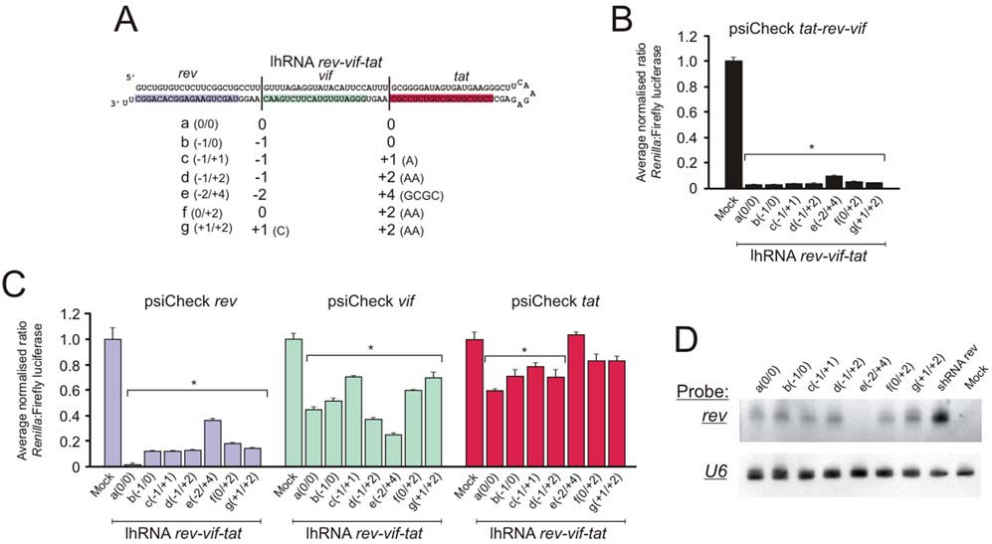
Effect of nucleotide spacing between *tat*, *rev* and *vif* siRNA-encoding sequences on silencing efficacy. A. Schematic illustration of hairpin sequences with boundaries between *tat*, *rev* and *vif* duplexes indicated. The sequences and numbers of bases inserted or deleted at the junctions of the RNAi effecter-encoding sequences are indicated for each of lhRNA *rev*-*vif*-*tat* a to lhRNA *rev*-*vif*-*tat* g. B. Average normalized *Renilla*∶Firefly luciferase activity determined 48 hours after transfecting HEK293 cells with the psiCheck *tat-rev-vif* target together with each of lhRNA *rev*-*vif*-*tat* a to lhRNA *rev*-*vif*-*tat* g. C. Average normalized *Renilla*∶Firefly luciferase activity determined 48 hours after transfecting HEK293 cells with the psiCheck *tat*, psiCheck *rev or* psiCheck *vif* target together with each of lhRNA *rev*-*vif*-*tat* a to lhRNA *rev*-*vif*-*tat* g. Results are given as the average values with standard deviations from three independent transfection experiments. (*, *p*<0.05, *t*-test, relative to mock transfected control). Mock transfected cells received the empty backbone U6+1 plasmid D. Northern blot analysis of RNA extracted from HEK293 cells that had been transfected with the indicated lhRNA and shRNA-expressing plasmids. The blot was probed with an oligonucleotide that was complementary to putative *rev* guide sequence. The blot was stripped and reprobed with an oligonucleotide complementary to U6 snRNA to control for equal RNA loading.

To assess the effect of the spacing between siRNA-encoding sequences on silencing efficiency, the lhRNA *rev-vif-tat* encoding cassette was modified by insertion or deletion of 1–4 bases at each of the junctions of the siRNA-encoding sequences (Fig. 4A). When these modified lhRNA *rev-vif-tat*-derived expression cassettes were transfected into HEK293 cells together with psiCheck *tat*-*rev*-*vif* target, the silencing efficacy of each of the RNAi effector sequences was similar, and approximately 95% silencing was achieved (Fig. 4B). Slightly diminished efficacy was observed for lhRNA *rev*-*vif*-*tat* e. This sequence had a deletion of 2 bp at the *rev*-*vif* junction, which may influence processing and silencing efficacy of the siRNA originating from the stem base. Assessment of silencing of individual *tat*, *rev* and *vif* targets again showed that the silencing was greatest for each target cognate of the siRNA derived from the stem base of the lhRNA sequence (Fig. 4C). When using the psiCheck *rev* target, knockdown of approximately 90% was achieved by all of the hairpins except for lhRNA *rev*-*vif*-*tat* e, which again showed diminished efficacy. No rev siRNA guide was observed for lhRNA *rev*-*vif*-*tat* e by northern blot when probing for rev (Fig. 4D), explaining the lack of inhibitory activity for this lhRNA species when detecting effects at the first position (for *rev*, Fig 3C). Diminished knockdown of reporter gene activity was observed when the fused target corresponded to the second position siRNA within the lhRNA duplex. However, the different spacing arrangements resulted in significant variation in silencing efficiency at this position. Interestingly, lhRNA *rev*-*vif*-*tat* e was more effective than each of the other lhRNA *rev*-*vif*-*tat* cassettes against the *vif* target. The 2 bp deletion at the *rev*-*vif* junction of lhRNA *rev*-*vif*-*tat* e may be the most optimal spacing arrangement for the *vif* siRNA sequence, which is in the second position. Inhibition of the reporter-*tat* target (third position) was largely ineffective for all of the lhRNA variants. The results suggest that there exists considerable leeway in improving first and second position siRNA arrangements along an lhRNA duplex but that third position siRNAs are unlikely to be dramatically improved by these modifications.

### Inhibition of HIV-1 replication in infected cells in culture

**Figure 5:**
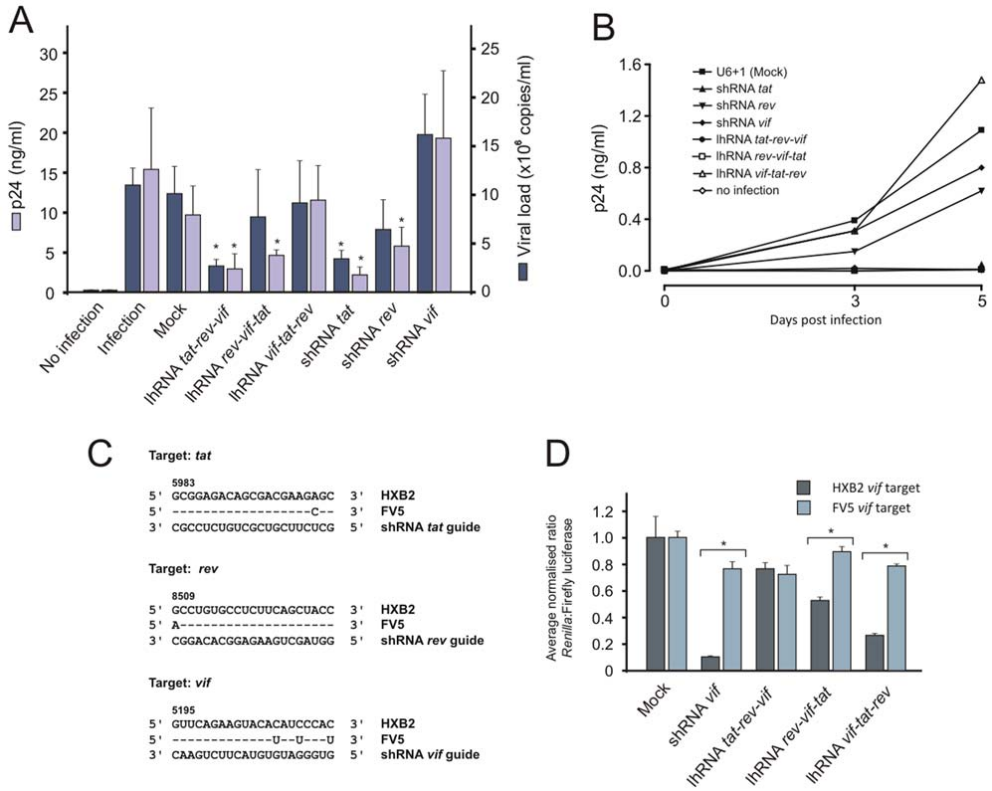
HIV-1 challenge assay. U87.CD_4_.CCR5 cells were transfected with plasmids expressing the indicated hairpins and then subjected to infection with an equivalent of TCID_50_ 1000 particles of the HIV-1 FV5 viral isolate. Concentrations of HIV-1 p24 in the culture supernatant and viral particle equivalents (A) were determined 6 days after infection. Results are expressed as the means with standard deviations of three independent experiments (*, *p*<0.05, *t*-test, relative to mock transfected control). B. Replication kinetics of a representative experiment included in (A). C. Sequence of lhRNA- and shRNA-derived *tat*, *rev* and *vif* guide sequences and complementary regions targeted within FV5 and HXB2 HIV-1 isolates. Mismatches between the putative guide and target sequences are shown. Sequence numbering is based on isolate HXB2, accession K03455. D. Knockdown of FV5 *vif* and HXB2 *vif* target-reporter fusion gene expression by shRNA *vif* and representative lhRNAs in HEK293 cells. Results are expressed as the means with standard deviations of three independent experiments (*; p<0.05, *t*-test, between annotated samples).

To assess the efficacy of lhRNA sequences in a culture model of HIV-1 infection, U87.CD_4_.CCR5 cells were transfected with various lhRNA expression plasmids followed by viral challenge with a South African R5-tropic subtype C HIV-1 isolate, FV5 (accession: 05ZAFV5). Knockdown was assessed by determining p24 antigen levels and viral RNA genome equivalents (Fig. 5A) in the culture supernatant at day 6 post-infection. Of the lhRNA expression cassettes, lhRNA *tat*-*rev*-*vif* was most effective and achieved inhibition of markers of viral replication by 60–70%. shRNA *tat* was the most effective of the shRNA expression cassettes and effected inhibition of approximately 60%. Both lhRNA *rev*-*vif*-*tat* and shRNA *rev* were less effective whereas lhRNA *vif*-*tat*-*rev* and shRNA *vif* respectively had weak or no inhibitory effect on HIV-1 replication in this cell culture model. The efficacy of silencing was also observed longitudinally, again indicating the ineffective silencing by shRNA *vif* and lhRNA *vif*-*tat*-*rev* (Fig. 5B) . Silencing by the second and third position siRNAs from lhRNA *vif-tat-rev* are not contributing to the inhibition of HIV-1 replication, but the first position is the most significant for the other 2 lhRNAs. Analysis of the sequences of the targets from the FV5 isolate (Fig. 5C) reveals that the putative hairpin-derived *vif* guide is not perfectly complementary to its viral cognate and includes 3 G∶U wobble mismatches. Although, shRNA vif was originally chosen as it proved to be effective at inhibiting viral progression and replication [34], it is possible that inhibiting *vif* may not immediately influence p24 output. To determine if the FV5 *vif* target sequence is refractory to silencing by shRNA *vif* and respective *vif*-containing lhRNAs, a psiCheck luciferase reporter vector was constructed containing HXB2 and FV5 *vif* shRNA target sequences. When compared to the inhibition of the HXB2 *vif* target, shRNA *vif* or lhRNA *vif-tat-rev* was unable to inhibit the FV5 target (Fig 5D). These data may explain why no viral inhibition was observed for shRNA *vif* and lhRNA *vif-tat-rev* (Figs 5A,B). The results of the challenge assay support the previous reporter knockdown data that lhRNA knockdown efficacy is primarily mediated by first position siRNA sequences located at the base of the duplex stem.

## Discussion

The lhRNAs designed in this study were developed specifically to generate three separate functional siRNAs targeting known non-adjacent siRNA-susceptible regions of HIV-1. Such an approach is of tremendous value to efforts aimed at combinatorial RNAi strategies, where the targeting of highly mutagenic sequences, such as HIV-1, may help prevent the emergence of resistant viral variants [12], [13]. Combinatorial RNAi approaches applied to date include the use of multiple RNA Pol III promoters to express shRNAs [15], [35], [36]. Various combinatorial shRNA-expression systems have been shown to delay effectively the emergence of shRNA-resistant HIV-1 in cell culture [15], [36], proving in principle the efficacy of a multiple targeting RNAi strategy against a rapidly evolving target. However, there are reservations about the use of multiple RNA Pol III expression cassettes. Firstly, little is known about the long-term stability and efficacy of such a system: adjacent repeat sequences may recombine when delivered by viral vectors or when stably expressed in rapidly dividing cells [36]. Secondly, and more importantly, the use of multiple highly active RNA Pol III promoters can potentially flood the cell with shRNAs and abrogate the natural microRNA biogenesis pathway, leading to unwanted toxicities [37]–[39]. It is clear that the presence of several therapeutic hairpin species will require careful dosing in order to achieve the desired levels of silencing.

To date, lhRNAs expressed from RNA Pol III promoters have been used against a single contiguous target sequence in mammalian cells. Previous reports targeting HCV and HIV have suggested that lhRNAs (>50 bp) can adequately inhibit targets harboring mutations that abrogate the silencing efficiency of 21 bp shRNAs [24], [30], [33]. Yet, the efficacy of individually processed siRNAs generated from expressed lhRNAs has not been adequately characterized, making it difficult for direct comparisons between lhRNAs and shRNA when targeted to the same sequence. We have previously observed that ∼60 bp U6-expressed lhRNAs targeted to a contiguous sequence within HBV generated siRNAs more efficiently from the base of the hairpin stem, and this correlated with greater silencing efficacy [26]. However, we could not rule-out the possibility that second or third Dicer cleavage reactions generate siRNAs with ineffective guide sequences. We suspect that an increased variance of the siRNA pool and decreased siRNA concentration for second and third position cleavage products is likely to compromise the efficacy of siRNAs generated from lhRNAs that require more than two Dicer reactions. By placing three known effective siRNA sequences adjacent to each other along a 69 bp lhRNA duplex, some general principles concerning the efficacy of expressed lhRNAs for combinatorial RNAi have been deduced. By comparing different combinations of adjacent siRNA sequences within a lhRNA duplex, we show that sequences at the base of the hairpin stem are preferentially processed into effective siRNAs, and that the pattern of silencing appears to be independent of the siRNA sequence within the duplex. There was an exception, as construct lhRNA *rev*-*vif*-*tat* e (Fig 4) was not able to generate a guide strand for the first position. Some sequence differences do exist between lhRNA *rev*-*vif*-*tat* e and other lhRNA variants, and it may be possible that the sequence of the 2 nt 3′ overhang plays a role in siRNA recognition within RISC. The Paz domain of Dicer is known to have biased preference for different 3′ overhang sequences [40], and differential selection of processed siRNAs by the analogous Argonaut 2 Paz domain may occur similarly [41], [42]. Overall, these data are in agreement with Dicer's preference for cleaving dsRNA duplex ends with 2-nt 3′-OH overhangs [40], [43]–[45] but suggests that intracellular Dicer processivity is relatively inefficient. This perhaps underscores the function of human Dicer as a single-turnover enzyme specialized in generating mature miRNAs from a single cleavage reaction.

Initially, the three independent siRNA-encoding sequences were placed within the lhRNA duplex such that they were spaced at 23 bp intervals. Recently, Liu et al [32] showed that extended shRNAs with two independent siRNAs functioned optimally as independent siRNAs when spaced 4 bp apart. However, in our hands, such spacing arrangements were not necessarily optimal and it remains difficult to make gross generalizations regarding Dicer-processing positions along an expressed dsRNA duplex at this stage. Nevertheless, improvements in multiple targeting can be achieved by further investigating the addition or deletion of nucleotides at the siRNA junctions along the duplex. For one of the lhRNAs, lhRNA *rev-vif-tat*, efficient processing of the second siRNA was observed, albeit at reduced concentrations. If arranged correctly, augmented knockdown can be achieved for two independent siRNAs along a duplex, but this unlikely to be possible for three siRNAs. Thus, one can envisage that use of lhRNAs designed to efficiently inhibit at least two independent siRNA-susceptible regions may help to delay the onset of HIV-1 escape variants, especially when targeting only conserved sequences [46]. It is unlikely that a third siRNA produced by Dicer cleavage of an lhRNA will be present in sufficient concentration to produce three effective siRNAs. We therefore provide a note of caution for the use of lhRNAs containing more than two adjacent siRNA sequences aimed at effective combinatorial RNAi. Nevertheless, lhRNAs in combination with other multiple RNAi effector sequences, such as Pol II-expressed multiple miRNA precursors [14], [18], [19], are likely to provide an effective means of targeting rapidly evolving sequences such as HIV-1.

In conclusion, we show that RNA Pol III-expressed lhRNAs are capable of producing independent siRNAs that induce significant knockdown of non-contiguous siRNA-susceptible regions of HIV-1. Importantly, the position and arrangement of the siRNA-encoding sequences along the lhRNA duplex plays an important role in determining the overall efficacy of the lhRNA in target suppression. Nonetheless, by optimizing the particular arrangement of siRNA-encoding sequences along the lhRNA duplex, effective multiple targeting is possible for up to two Dicer processing reactions. We therefore provide a useful framework for investigating the use of RNA Pol III-expressed lhRNAs aimed at effective combinatorial RNAi in mammalian cells.

## Materials and Methods

### Target plasmids

The psiCheck target plasmids were prepared by directed insertion of the *Xho*I-*Not*I digested HIV-1 PCR fragments into the plasmid psiCheck2 (Promega, WI, USA) such that the target sequences were within the 3′ UTR of *Renilla* Luciferase. The individual shRNA target sequences were amplified by PCR from pNL4-3 template [47] using the following primers: sh *tat* target F 5′- GAT CTC GAG AGT GTT GCT TTC ATT GCC AA-3′ (29 nt), sh *tat* target R 5′-GAT CGC GGC CGC GCA TTA CAT GTA CTA CTT ACT GCT T-3′ (37 nt). sh *rev* target F 5′-GAT CTC GAG AAG GTG GAG AGA GAG ACA GA-3′ (29 nt), sh *rev* target R 5′-GAT CGC GGC CGC CAC CAA TAT TTG AGG GCT TC-3′ (32 nt). sh *vif* target F 5′-GAT CTC GAG ATT TCA AGG AAA GCT AAG GA-3′ (29 nt), sh *vif* target R 5′-GAT CGC GGC CGC AAT GCC AGT CTC TTT CTC CT-3′ (32 nt). To generate a product consisting of all three target sites adjacent to one another, complementary oligonucleotides were treated with polynucleotide kinase (Promega, WI, USA), annealed, and cloned directly into the XhoI-NotI sites of psiCheck2. To facilitate screening, an EcoRV site was inserted within each annealed dsDNA insert. The oligonucleotides used include: target *tat-rev-vif* (+) 5′- GAT CTC GAG GCG GAG ACA GCG ACG AAG AGC TTG CCT GTG CCT CTT CAG CTA CC-3′ (53 nt) and target *tat-rev-vif* (−) 5′-GAT CGC GGC CGC GTG GGA TGT GTA CTT CTG AAC AAG GTA GCT GAA GAG GCA CAG GC-3′ (58 nt). Similarly, psiCheck clones were constructed for a HXB2 and FV5 *vif* target: target FV5-*vif* (+) 5′-TCG AGA TAT CGT TCA GAA GTA CAT ATT CCA TGC -3′ and target FV5-*vif* (−) 5′-GGC CGC ATG GAA TAT GTA CTT CTG AAC GAT ATC -3′; target HXB2-*vif* (+) 5′-TCG AGA TAT CGT TCA GAA GTA CAC ATC CCA CGC -3′ and target HXB2-*vif* (−) 5′-GGC CGC GTG GGA TGT GTA CTT CTG AAC GAT ATC -3′.

### Long hairpin RNA and short hairpin RNA expression plasmids

The procedure for generating Pol III U6 shRNA cassettes has been previously described [4], [48]. A similar 2 step PCR approach was used to produce the lhRNA and shRNA vectors complementary to the HIV-1 *vif*, *tat*, and *rev* genes. The first amplification was carried out with a universal U6 forward primer and first lhRNA or shRNA reverse primer with U6 promoter plasmid DNA as template. The amplified product was used as template for a PCR step with a second lhRNA or shRNA reverse primer and again the universal U6 forward primer. The sequence of the U6 universal forward primer was 5′- CTA ACT AGT GGC GCG CCA AGG TCG GGC AGG AAG AGG G-3′. Sequences of the reverse primers for the amplifications were as follows: lhRNA *tat-rev-vif* R1 5′- CTT GAA ATG GAA TGT ATA CCT CTA AAC AAG GCA GCC GAA GAG ACA CAG ACA AGC CCT TCA TCA CTA TCC CCG CGG TGT TTC GTC CTT TCC ACA A -3′ (94 nt), lhRNA *tat-rev-vif* R2 5′- AAA AAA GCG GAG ACA GCG ACG AAG AGC TTG CCT GTG CCT CTT CAG CTA CCT TGT TCA GAA GTA CAC ATC CCA CTC TCT TGA AAT GGA ATG TAT A -3′ (94 nt). lhRNA *rev-vif-tat* R1 5′- CTT GAA GCC CTT CAT CAC TAT CCC CGC AAA TGG AAT GTA TAC CTC TAA ACA AGG CAG CCG AAG AGA CAC AGA CGG TGT TTC GTC CTT TCC ACA A -3′ (94 nt), lhRNA *rev-vif-tat* R2 5′- AAA AAA GCC TGT GCC TCT TCA GCT ACC TTG TTC AGA AGT ACA CAT CCC ACT TGC GGA GAC AGC GAC GAA GAG CTC TCT TGA AGC CCT TCA TCA C -3′ (94 nt). lhRNA *vif-tat-rev* R1 5′- CTT GAA GGC AGC CGA AGA GAC ACA GAC AAG CCC TTC ATC ACT ATC CCC GCA AAT GGA ATG TAT ACC TCT AAA CGG TGT TTC GTC CTT TCC ACA A -3′ (94 nt), lhRNA *vif-tat-rev* R2 5′- AAA AAA GTT CAG AAG TAC ACA TCC CAC TTG CGG AGA CAG CGA CGA AGA GCT TGC CTG TGC CTC TTC AGC TAC CTC TCT TGA AGG CAG CCG AAG A -3′ (94 nt). shRNA *tat* R1 5′- CTC TTG AAG CCC TTC ATC ACT ATC CCC GCG GTG TTT CGT CCT TTC CAC AA -3′ (50 nt), shRNA *tat* R2 5′- AAA AAA GCG GAG ACA GCG ACG AAG AGC TCT CTT GAA GCC CTT CAT CAC -3′ (48 nt). shRNA *rev* R1 5′- CTC TTG AAG GCA GCC GAA GAG ACA CAG ACG GTG TTT CGT CCT TTC CAC AA -3′ (50 nt), shRNA *rev* R2 5′- AAA AAA GCC TGT GCC TCT TCA GCT ACC TCT CTT GAA GGC AGC CGA AGA -3′ (48 nt). shRNA *vif* R1 5′- CTC TTG AAA TGG AAT TGT ATA CCT CTA AAC GGT GTT TCG TCC TTT CCA CAA -3′ (50 nt), shRNA *vif* R2 5′- AAA AAA GTT CAG AAG TAC ACA TCC CAC TCT CTT GAA ATG GAA TGT ATA -3′ (48 nt). The sequences for variants of lhRNA *rev-vif-tat* were: lhRNA *rev-vif-tat* b R1 5′- CTC TTG AAG CCC TTC ATC ACT ATC CCC GCA AAT GGA ATG TAT ACC TCT AAA CAG GCA GCC GAA GAG ACA CAG ACG GTG TTT CGT CCT TTC CAC AA-3′ (95 nt), lhRNA *rev-vif-tat* b R2 5′- AAA AAA GCC TGT GCC TCT TCA GCT ACC TGT TCA GAA GTA CAC ATC CCA CTT GCG GAG ACA GCG ACG AAG AGC TCT CTT GAA GCC CTT CAT C-3′ (91 nt). lhRNA *rev-vif-tat* c R1 5′- CTC TTG AAG CCC TTC ATC ACT ATC CCC GCT AAA TGG AAT GTA TAC CTC TAA ACA GGC AGC CGA AGA GAC ACA GAC GGT GTT TCG TCC TTT CCA CAA -3′ (96 nt), lhRNA *rev-vif-tat* c R2 5′- AAA AAA GCC TGT GCC TCT TCA GCT ACC TGT TCA GAA GTA CAC ATC CCA CTT AGC GGA GAC AGC GAC GAA GAG CTC TCT TGA AGC CCT TCA TCA -3′ (93 nt). lhRNA *rev-vif-tat* d R1 5′- CTC TTG AAG CCC TTC ATC ACT ATC CCC GCT TAA ATG GAA TGT ATA CCT CTA AAC AGG CAG CCG AAG AGA CAC AGA CGG TGT TTC GTC CTT TCC ACA A -3′ (97 nt), lhRNA *rev-vif-tat* d R2 5′- AAA AAA GCC TGT GCC TCT TCA GCT ACC TGT TCA GAA GTA CAC ATC CCA CTT AAG CGG AGA CAG CGA CGA AGA GCT CTC TTG AAG CCC TTC ATC A -3′ (94 nt). lhRNA *rev-vif-tat* e R1 5′- CTT GAA GCC CTT CAT CAC TAT CCC CGC GCG CAA ATG GAA TGT ATA CCT CTA AAC GGC AGC CGA AGA GAC ACA GAC GGT GTT TCG TCC TTT CCA CAA -3′ (96 nt), lhRNA *rev-vif-tat* e R2 5′- AAA AAA GCC TGT GCC TCT TCA GCT ACC GTT CAG AAG TAC ACA TCC CAC TTG CGC GCG GAG ACA GCG ACG AAG AGC TCT CTT GAA GCC CTT CAT CAC -3′ (96 nt). lhRNA *rev-vif-tat* f R1 5′- CTT GAA GCC CTT CAT CAC TAT CCC CGC TTA AAT GGA ATG TAT ACC TCT AAA CAA GGC AGC CGA AGA GAC ACA GAC GGT GTT TCG TCC TTT CCA CAA -3′ (96 nt), lhRNA *rev-vif-tat* f R2 5′- AAA AAA GCC TGT GCC TCT TCA GCT ACC TTG TTC AGA AGT ACA CAT CCC ACT TAA GCG GAG ACA GCG ACG AAG AGC TCT CTT GAA GCC CTT CAT CAC -3′ (96 nt). lhRNA *rev-vif-tat* g R1 5′- CTT GAA GCC CTT CAT CAC TAT CCC CGC TTA AAT GGA ATG TAT ACC TCT AAA CGA AGG CAG CCG AAG AGA CAC AGA CGG TGT TTC GTC CTT TCC ACA A -3′ (97 nt), lhRNA *rev-vif-tat* g R2 5′- AAA AAA GCC TGT GCC TCT TCA GCT ACC TTC GTT CAG AAG TAC ACA TCC CAC TTA AGC GGA GAC AGC GAC GAA GAG CTC TCT TGA AGC CCT TCA TCA C -3′ (97 nt). The 63 bp lhRNA control plasmid, lhRNA TAR, which was designed to target an irrelevant site which included the HIV-1 TAR stem-loop (complementary to positions 454–512, numbering according to HIV-1 HXB2 sequence, accession K03455), has been previously described [26]. For all lhRNA constructs, each pair of primers had an overlapping sequence of 19 bases that enabled extension of the PCR product to generate a U6 promoter lhRNA cassette with a RNA Pol III transcription termination signal [48]. Amplified PCR products were ligated to a T/A cloning vector (pTZ57R/T, Fermentas, WI, USA) to generate pTZ-U6 lhRNA and shRNA plasmids. Sequences were confirmed by standard procedures.

### Cell culture

The human embryonic kidney cell line, HEK293, was maintained in Dulbecco's Modified Eagle's Medium (DMEM, BioWhittaker, MD, USA) supplemented with 10% heat inactivated fetal calf serum (FCS, Delta Bioproducts, Johannesburg, SA) at 37°C and 5% CO_2_. The human astrocyte glioblastoma cell line, U87.CD_4_.CCR5 (NIH AIDS Research and Reference Reagent Program), was maintained in DMEM supplemented with 15% heat inactivated FCS, 50 IU/mL Penicillin/50 µg/mL Streptomycin mix (Gibco, BRL, UK), 1 µg/mL Puromycin (Merck, London, UK), 300 µg/mL G418 (Sigma, MO, USA) and 1% L-glutamine (Sigma, MO, USA) at 37°C and 5% CO_2_.

### Transfections

Transfections were carried out using a ratio of 1 µL Lipofectamine2000 (Invitrogen, CA, USA) to 1 µg total DNA per well according to the manufacturer's instructions. Media was changed 24 hours post transfection, and analysis of cells was carried out 24 hours thereafter. Equivalent transfection efficiencies were verified by fluorescence microscopy by cotransfecting a plasmid that constitutively produces enhanced green fluorescent protein (pCI-eGFP) [49].

To evaluate the effects of the lhRNA and shRNA encoding plasmids on a reporter target, HEK293 cells were seeded 24 hours prior to transfection at 120 000 cells per well in 24 well culture dishes. HEK293 cells were transfected with 150 ng of target plasmid, 750 ng of lhRNA or shRNA encoding plasmid and 100 ng of pCI-eGFP.

To determine the induction of IFN response-related genes, HEK293 cells were seeded as described above and transfected with 900 ng of lhRNA or shRNA encoding plasmid and 100 ng pCI-eGFP per well. Control double stranded RNA, poly (I∶C) (Sigma, MO, USA), was transfected at equivalent amounts to the hairpin encoding plasmids.

For Northern blot analysis HEK293 cells were seeded at 80% confluency in 10 cm culture dishes 24 hours prior to transfection. Cells were transfected using Lipofectamine with 16 µg of lhRNA or shRNA encoding plasmid, 3 µg of target plasmid, and 1 µg pCI-eGFP.

To assess the effects of the lhRNA encoding plasmids on a subtype C HIV-1 primary isolate in an infection challenge assay, U87.CD_4_.CCR5 cells (NIH HIV/AIDS Reagent and Reference Program) were washed with 1×PBS, treated for 5 minutes with 1×trypsin, counted as described above and seeded 24 hours prior to transfection at 100 000 cells per well in 12 well culture dishes using DMEM supplemented with 15% heat inactivated FCS only. The following day, cells were co-transfected with 900 ng of lhRNA encoding plasmid and 100 ng of pCI-GFP per well as described above.

### Dual luciferase assay

These were carried out according to the manufacturer's instructions (Promega, WI, USA) using a Veritas dual-injection luminometer (Turner Biosystems, C A, USA). Target-specific *Renilla* luciferase expression was normalized to background firefly luciferase expression. Average expression ratios for a control plasmid containing the U6 promoter was set to 100%, and relative expression levels for other samples calculated accordingly. Two independent experiments in triplicate were performed and the data are expressed as the mean±standard deviation.

### Viral propagation and challenge assay

FV5 is a primary HIV-1 CCR5-utilizing subtype C virus that was isolated from a drug-naïve HIV-positive AIDS patient admitted to the Johannesburg Hospital AIDS clinic, and propagated by standard PBMC co-culture techniques. The co-receptor tropism of FV5 was established genotypically by automated sequencing of the V3 loop of the viral *env* gene (accession 05ZAFV5), and confirmed phenotypically by MT-2 fusion assay. Twenty four hours post-transfection, U87.CD_4_.CCR5 cells were infected with FV5 using a TCID_50_ 1000. Twenty four hours post infection cells were washed three times using 1×PBS and fresh media was added. At days 0 (day of washing), 3, 5 and 6, 100 µL of supernatant was collected per well and analysed by ELISA (Murex Biotech LTD, Dartford, UK) for p24 antigen production as a marker of viral replication. Viral RNA was extracted from 300 µL of day 6 supernatant using the COBAS Ampliprep instrument (Roche, Germany), followed by a viral load assay with the COBAS Amplicor (Roche, Germany) according to manufacturer's specifications. Day 0 p24 data was completed and in all cases no viral p24 protein or RNA was detected indicating that all residual infecting virus had been removed from the cultures.

### Northern blot analysis

Total RNA was extracted from HEK293 cells using TriReagent™ (Sigma, MO, USA) according the manufacturer's instructions 48 hours post-transfection. Twenty-five micrograms of RNA was resolved on urea denaturing 15% polyacrylamide gels and blotted onto nylon membranes. RNA molecular weight markers, which were radioactively labeled as described below, were run alongside the cellular RNA. Blots were hybridized to three DNA oligonucleotides (probes *tat*, *rev* and *vif*) to detect products of hairpin processing. These were complimentary to regions spanning the antisense sequence of the long hairpin. Probes were labeled at their 5′ ends with [*γ*-^32^ P] ATP and T4 polynucleotide kinase. After purification using standard procedures, they were hybridized to immobilized RNA, exposed to X-ray film and then stripped and reprobed. An oligonucleotide sequence complementary to U6 small nuclear RNA was used as a control to verify equal loading of the cellular RNA. Probe oligonucleotide sequences were as follows: probe *tat*: 5′-GCG GAG ACA GCG ACG AAG AGC TT-3′; probe *rev*: 5′-GCC TGT GCC TCT TCA GCT ACC TT-3′; probe *vif*: 5′-GTT CAG AAG TAC ACA TCC CAC TT-3′; and U6 small nuclear RNA probe: 5′-TAG TAT ATG TGC TGC CGA AGC GAG CA-3′. The LNA probe sequences were as follows: probe *LNA-tat-1*: 5′-ACT TGC GGA GAC AG-3′; probe *LNA-tat-2*: 5′-GCG CGC GGA GAC AG-3′. The LNA nucleotides are underlined.

### Statistics

Statistical calculations were determined using the GraphPad Prism software package (GraphPad, Software, Inc., CA, USA). Statistical difference was considered significant when *p*<0.05 and was determined using either an unpaired Student's *t*-test or by ANOVA.
